# Blood pressure and proteinuria control remains a challenge in patients with type 2 diabetes mellitus and chronic kidney disease: experience from the prospective observational ALICE-PROTECT study

**DOI:** 10.1186/s12882-016-0336-1

**Published:** 2016-09-21

**Authors:** Jean-Michel Halimi, Dominique Joly, Christian Combe, Gabriel Choukroun, Bertrand Dussol, Jean-Pierre Fauvel, Stéphane Quéré, Béatrice Fiquet

**Affiliations:** 1 Université François-Rabelais, Faculté de Médecine, Service de Néphrologie-Immunologie Clinique, Hôpital Bretonneau, CHU Tours and EA4245, Tours, France; 2 Université Paris-Descartes, Faculté de Médecine, AP-HP; Service de Néphrologie, Hôpital Necker-Enfants Malades, Paris, France; 3 Université Bordeaux Segalen, Service de Néphrologie Transplantation Dialyse, CHU de Bordeaux and INSERM U1026, Bordeaux, France; 4 Université de Picardie Jules Verne, Département de Néphrologie Dialyse Transplantation, CHU Amiens et INSERN UMR 1088, Amiens, France; 5 Aix-Marseille Université, Faculté de Médecine, Centre de Néphrologie et de Transplantation Rénale, Hôpital de la Conception, Marseille, France; 6 Université Claude Bernard Lyon, Department of Nephrology-Hypertension, Génomique Fonctionnelle de l’Hypertension artérielle, EA 4173, Hôpital Nord-Ouest, Villefranche sur Saône, Hospices Civils de Lyon, Lyon, France; 7 Clinical Affairs, Biostatistics, Clinical Research and Development, Novartis Pharma SAS, Rueil-Malmaison, France

**Keywords:** Type 2 diabetes mellitus, Nephropathy, Blood pressure, Proteinuria, Cardiovascular events, End stage renal disease, ALICE Protect study

## Abstract

**Background:**

Type 2 diabetes (T2DM) is the leading cause of chronic kidney disease (CKD) in western countries. The combination of both increases the risk of end stage renal disease (ESRD), cardiovascular events and all-cause mortality. Early control of blood pressure (BP) and proteinuria (Pu) is crucial to slow down the progression of the CKD and prevent cardiovascular events and mortality. The primary objective of the study was to assess BP and Pu control after a 2-year follow-up in T2DM patients with CKD.

**Methods:**

Prospective, multicenter, observational study. Overall, 153 French nephrologists included 986 T2DM patients with Pu (≥0.5 g/day) and an eGFR >15 ml/min/1.73 m^2^. Data from 729 patients were available after a 2-year follow-up. BP and Pu control were respectively defined as less than 140/90 mmHg and 0.5 g/day. We also looked at renal and cardiovascular events.

**Results:**

At baseline, 74 % of the patients were male, mean age was 70 years. The mean T2DM duration was 17 years with a mean HbA1c of 7.4 %. All were treated for hypertension and 33 % had a controlled BP; 81 % had dyslipidemia and LDLc was <1 g/L for 54 %; 44 % had retinopathy, 40 % macrovascular complications and 12 % heart failure. Mean Pu was 2 g/day and eGFR 40 ± 20 mL/min/1.73 m^2^, with 13, 18, 32 and 37 % of the patients in respectively stage 2, 3a, 3b and 4 CKD.

After two years, 21 % reached the Pu target and 39 % the BP target. The mean eGFR of 40 ± 20.3 ml/min/1.73 m^2^ at baseline dropped to 33.9 ± 22.6 ml/min/1.73 m^2^ by year two (*p* < 0.001). This corresponded to a mean annual eGFR reduction of 3.2 ml/min/1.73 m^2^. 118 patients presented a renal event (16.2 %): doubling of serum creatinine for 86 patients (11.8 %) and start of dialysis for 72 (9.9 %); 176 patients (24.1 %) developed at least one cardiovascular complication (mainly coronary events and acute heart failure) during the follow-up period, and among these, 50 had also developed renal complications. Sixty patients died, i.e., 8.2 %, 26 patients from cardiovascular causes.

**Conclusion:**

Our study highlights that achieving BP and Pu targets remains a major challenge in patients with T2DM and nephropathy. Renal failure emerges as a more frequent event than death.

## Background

Type 2 diabetes mellitus (T2DM) is a chronic progressive disease affecting over 370 million patients worldwide in 2012 [1]. In France, the prevalence of T2DM has been estimated to be around 5.5 % in 2012 and this figure is constantly rising as obesity and ageing of the population progress [2]. Diabetes-related complications are less frequent today than 20 years ago and this is mainly due to a better management of cardiovascular (CV) risk factors and improved glucose control [3]. While the largest decline was seen in rate of myocardial infarction (MI), the smallest reduction has been observed for end-stage renal disease (ESRD) and T2DM remains the leading cause of chronic kidney disease (CKD) in western countries [4] with a prevalence of 25 to 50 % worldwide [5–8] and almost 25 to 30 % in France [4, 9]. Data from a recently published French registry showed that ESRD related to T2DM increased from 2007 to 2011 with an absolute change of 21 %, only partially attributed to population ageing and increased prevalence of T2DM [9].

The combination of T2DM and CKD not only increases the risk of ESRD but also of CV events and all-cause mortality, with CV complications being the main cause of death in these patients [10]. Recently it has been shown that the risk of both MI and all-cause death in people with T2DM and CKD were similar to or higher than those in people with history of MI [11].

Two large cohorts have described the natural history of T2DM-associated nephropathy: the British UKPDS and the Steno center’s Danish study [12, 13]. Both studies showed that the development of nephropathy led to an increased risk of death, especially due to CV causes, and that the risk of death was also higher than the risk of renal disease progression.

Early detection and management of these patients are crucial and involve a close monitoring of CV risk factors, control of blood pressure (BP) and proteinuria (Pu) and use of drugs acting on the renin angiotensin system (RAS) to slow down the progression of the renal disease and prevent CV events and mortality [14–17]. Some debate remains as to the BP target to obtain: historically <130/80 mmHg [18], or more recently <140/85 mmHg [19], <140/80 mmHg [20] or <140/90 mmHg [21–23]. It is widely accepted that Pu should be decreased under 0.5 g/day [18].

Nevertheless, few real-life prospective studies report the proportion of T2DM patients with hypertension and Pu in whom the BP and Pu goals have been achieved and of the incidence of renal and CV events. In this context, we conducted the ALICE- PROTECT observational study.

## Methods

This is the final analysis of the 2-year follow-up of the French ALICE-PROTECT cohort which was a multicenter, observational, prospective study. Details of the cohort creation, the included patients and the data recorded are described elsewhere [24]. Briefly, adult outpatients with T2DM, clinical Pu [defined by 24-h Pu ≥ 0.5 g per day or urinary protein/creatinine ratio (UPCR) ≥ 50 mg/mmol (≥500 mg/g) or urinary albumin/creatinine ratio ≥ 30 mg/mmol (≥ 300 mg/g)] and an estimated glomerular filtration rate (eGFR by MDRD formula) over 15 mL/min/1.73 m^2^ were recruited by 153 active nephrologists throughout France between January 2010 and February 2011. The planned follow-up for the whole population was 2 years, up to June 2013, with one planned visit per year ± 4 months. No specific assessment was requested for the study.

### Assessments

The main goal of the ALICE-protect study was to evaluate the percentage of patients with T2DM and nephropathy reaching BP (< 130/80 mmHg) and Pu (< 0.5 g/day) targets after a 2-year follow-up in real-life conditions. Given that some European and French recommendations [19, 22] for the BP target to reach for T2DM patients or those with CKD changed during the course of the study, the same evaluation was also carried out using the threshold of BP < 140/90 mmHg.

Secondary endpoints were the occurrence of renal (doubling of serum creatinine and/or end- stage renal disease) and CV (acute coronary syndrome, stroke, amputation, hospitalization for heart failure, carotid, coronary and lower limb arteries revascularization) complications in real-life conditions. We also looked at the predictive factors for complications and reaching the BP and Pu goals.

### Data analysis-statistics

The statistical analysis plan was defined before the start of the analysis. Descriptive statistics of recruited patient characteristics were performed. Quantitative variables were described in terms of mean, standard deviation, median and outliers, and qualitative variables in terms of absolute frequency and percentage per modality. Depending on the nature of the variables (continuous or discrete) and data availability, t-tests or Wilcoxon tests and chi-squared or Fisher’s exact tests were carried out. To evaluate the statistical significance of changes to continuous parameters over time, Wilcoxon signed-rank tests were also adjusted.

Multivariate models were also used to determine the predictive factors for BP and Pu control and incidence of CV and renal events. For each event of interest, the relationship with potentially related factors was evaluated using univariate analysis, and then the significant factors at a threshold of *p* < 0.10 were entered into a logistic regression using a stepwise method and a maintenance threshold in the model set at *p* < 0.05. The factors included in the final model are described in the results with the Odds-Ratio (OR), their 95 % confidence interval (CI), and their *p*-values.

Three analyses were performed: data analysis of the initial questionnaire and the general questionnaire for nephrologists; analysis after the 1-year database lock; and then after the 2 year follow-up.

All statistical analyses were performed using SAS 9.3 software (SAS Institute, Cary, NC, USA). Details about sample size calculation have been published elsewhere [24].

## Results

### Demographic and disease characteristics of the population at baseline

Nine hundred eighty-six patients were recruited and analysed at baseline [24]. Of these, 630 patients were followed for 2 years (mean follow-up 23 ± 2.4 months) with clinical and biological data available.

**Fig. 1:**
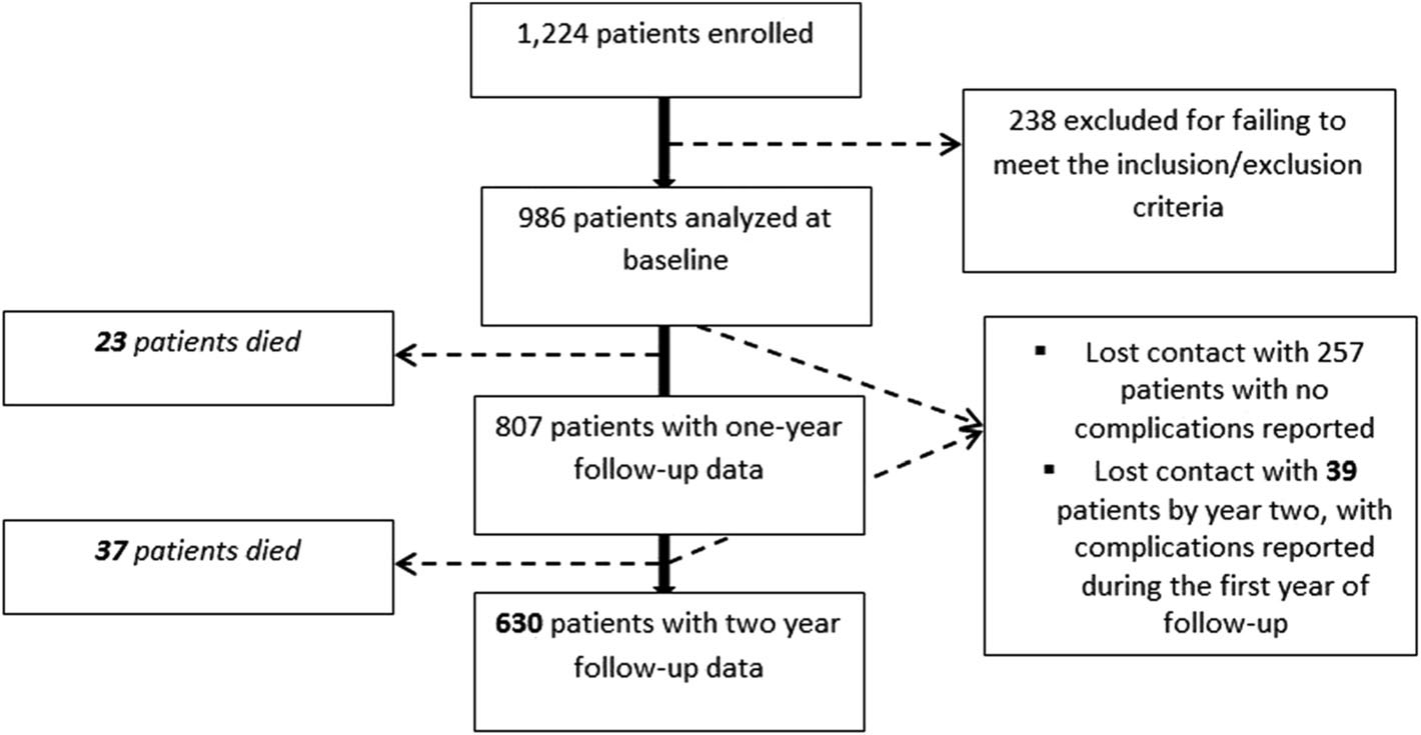
Disposition of the patients

These patients therefore constitute the analysed population for the study’s primary objective (BP and Pu control after a 2-year follow-up). The occurrence of CV or renal events was analysed in this population as well as in those patients who had died during the study or had been lost to follow-up by year two but who had experienced complications during the first year, i.e., 729 patients (cf. numbers in bold in Fig. 1).

Patients characteristics (*n* = 729) were not different from those described in the 986 analysed at baseline: mean age was 69 years with 31 % of the patients over 75 years, 74 % were male, mean body mass index (BMI) was 30 kg/m^2^ and most patients had a long history of diabetes (mean duration of 17 years). The mean HbA1c was 7.4 %: 43 % of the patients had an HbA1c <7 % and 25 % had an HbA1c ≥8 %. Essentially all received an antidiabetic treatment (98 %), mostly insulin alone or combined with oral antidiabetic agents (Table 1). Nearly all patients had at least two associated CV risk factors: all had hypertension (mean office BP was 149 ± 20 / 79 ± 11 mmHg 33 % with controlled BP i.e., < 140/90 mmHg), 10 % were active smokers, 81 % had dyslipidemia. Details on antihypertensive and statin treatments are presented in Table 1. Most patients received antihypertensive treatments (99.3 %) and 75 % were treated with three antihypertensive agents or more.

**Table 1:** Treatments at baseline and at year 2

	Baseline *N* = 729	Visit at year 2 *N* = 688
*Antidiabetic treatment* (% of the treated patients)		
Insulin ± OAD^a^	62.5	72.3
OAD only	37.5	27.7
*Antihypertensive treatment* (% of the treated patients)		
RAS blockers (at least one)	92.1	86.5
ARBs	62.8	59.4
ACEi	39.4	35.9
DRI	12	8.5
Diuretics (at least one)	77.1	80.4
Loop diuretic	54.4	61.2
Thiazides	29	27.5
Antialdosterone	3.2	3.5
CCB	67.5	63.9
Beta-blockers	50.3	56.5
Central acting agents and others	25.7	27.4
*Other treatments* (% of the patients)		
Statins	76.4	69.6
Antiplatelet therapy	61.3	55.7

^a^Oral Antidiabetic Agents

**Fig. 2:**
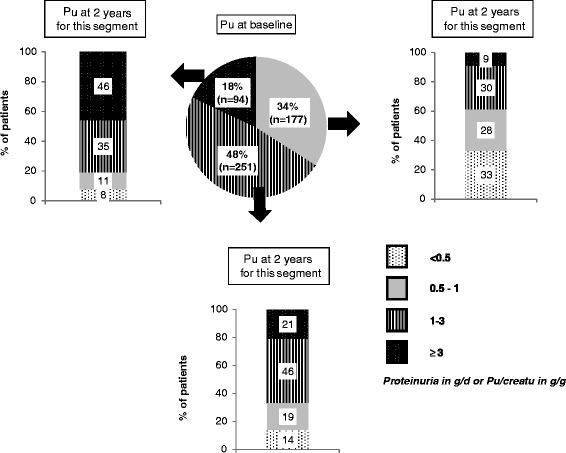
Change in Pu stage at the end of the study based on the Pu stage recorded at baseline

As required by the inclusion criteria, all patients had nephropathy. Other diabetic complications, were highly prevalent driven by retinopathy (44 %), macrovascular complications (40 %, mainly coronary heart disease for 27 % of the patients), and symptomatic heart failure (12 %). Mean eGFR was 40 mL/min/1,73 m^2^: 37 % of the patients had stage 4 CKD (eGFR < 30 mL/min/1,73 m^2^), 50 % had stage 3 CKD (eGFR 30–60 mL/min/1,73 m^2^), 10 % stage 2 CKD (eGFR 60–90 mL/min/1,73 m^2^), and 3 % had normal eGFR. The mean Pu was 1.9 ± 1.7 g/day (median 1.3 g/day, 25–75 % quartiles [0.8; 2.3]), 34 % of the patients had Pu between 0.5 and 1 g/day or an UPCR between 0.5 and 1 g/g, 48 % between 1 and 3 g/day ou g/g and 18 % above 3 g/day ou g/g (Fig. 2). Overall, 91.5 % of the patients were on RAS blockers [angiotensin conversion enzyme inhibitors (ACEi), angiotensin receptor blockers (ARB) or direct renin inhibitor (DRI)]: 70 % with only one and 21.5 % with two.

The 257 patients lost to follow-up were similar at baseline from the above population of 729 patients: the sex ratio was the same, as was the mean age and duration of diabetes. They presented with similar CV risk factors and diabetes-related complications, with the only exception being a slightly lower incidence of retinopathy (37 % compared to 44 %, *p* = 0.053). Although their Pu rates were similar, they had slightly higher eGFR values (44.5 vs 40 ml/min/1.73 m^2^, *p* = 0.018). The treatments they were receiving were not overly different, notably with a similar proportion of patients with RAS blockers (around 91 %), although dual RAS blockade was more frequent in the patients who were lost to follow-up (28.8 % compared to 21.5 %, *p* = 0.017).

### Blood pressure and proteinuria control after 2 years

The dual objective of BP < 130/80 mmHg and Pu < 0.5 g/d or UPCR < 0.5 g/g was achieved for 25 patients (4.5 %) out of the 558 patients in whom the BP and Pu values (24-h measurement or UPCR) were available at 2 years.

As mentioned in the methods section, the same evaluation was carried out using the threshold of BP < 140/90 mmHg: 62 patients (11.1 %) achieved the dual objective.

### Blood pressure

Mean systolic BP (SBP) decreased significantly from 148.5 ± 20.5 mmHg at baseline to 144 ± 19.7 mmHg at year two, given an average reduction of 4.5 ± 22.9 mmHg (*p* < 0.001). A similar change was observed in average diastolic blood pressure (DBP) (78.8 ± 11.3 mmHg at baseline), with a significant reduction of 2.8 ± 12.9 mmHg at year two (*p* < 0.001). After the 2-year follow-up period, 14.3 % of patients had a BP < 130/80 mmHg and 38.8 % had a BP < 140/90 mmHg. Home BP monitoring (HBPM) or ambulatory BP monitoring (ABPM) values were reported for only 8.6 and 1.3 % of the followed patients respectively.

The number of patients who didn’t receive any antihypertensive treatment increased from 0.7 % at baseline to 9.7 % after 2 years. The number of patients receiving a four-drug therapy or more decreased from 46 % at baseline to 39 % after 2 years. Overall, antihypertensive treatment score remained constant in 39 % and decreased in 34 % of the patients with uncontrolled BP at year two.

At year 2, the different antihypertensive classes used were similar to those at baseline (Table 1). Different diuretics were combined in 14.6 % of the diuretic treated patients at the end of the study.

Controlled BP at baseline, the lowest Pu rate at year two and the absence of diuretic treatment at year two were significant factors associated with reaching the BP target of < 140/90 mmHg in the multivariate analysis (Fig. 4a).

### Proteinuria

Pu was reported by using 24-h urine measurement in 384 patients, by an UPCR in 202 patients, and by Pu concentration alone in 99 patients. The changes over time were evaluable in 522 patients either by 24-h Pu readings or UPCR.

The mean 24-h Pu and UPCR remained stable throughout the study at 1.9 ± 1.7 g/day and 2.0 ± 2.6 g/g respectively (measurement at the end of the study). At the end of the study, 20 % of patients had reached the Pu target (24-h quantitation or UPCR) of < 0.5 g/day or g/g, 21 % had Pu between 0.5 and 1 g/day or UPCR between 0.5 and 1 g/g, 38%between 1 and 3 g/day or UPCR between 1 and 3 g/g and 21 % of patients had nephrotic range Pu. This latter group was mainly composed of patients who had nephrotic range Pu at baseline, however a marked increase of Pu, from < 1 g/day to > 3 g/day was observed in 2.6 % of the patients overall (Fig. 2).

The number of patients receiving at least one RAS blocker (ARBs and/or ACEi and/or DRI) fell during the course of the study, from 91.5 % of patients at baseline to 78.1 % at year two. Among these patients, single RAS blockade was the most common: 76.5 % at baseline and 80 % at year two. Dual RAS blockade was prescribed for 23 % of patients at baseline and 20 % at year two; the use of triple RAS blockade was marginal, concerning three patients at baseline and none by the end of the study. The preferred combination was an ARB and an ACEi, and this trend was even more marked at the end of the study, as the proportion of ACEi + ARB combinations used in patients receiving a dual RAS blockade rose from 69.5 % at baseline to 87 % by year two. Among the patients who were receiving a single RAS blocker at baseline, at year two most remained with a single RAS blockade (72 %), less than 9 % received a dual RAS blockade, and almost 20 % were not receiving any RAS blocking agents. Among the patients who were receiving a dual RAS blocker at baseline, at year two 45 % were still on this dual blockade, 40 % were given a single RAS blocker, and 15 % were no longer receiving any RAS blockers.

Pu levels after the 2-year follow-up had a moderate impact on the prescribed RAS blocker treatment. Therefore, in patients with a Pu > 1 g/day or an UPCR > 1 g/g, 20 % were not receiving any RAS blockers at year two, 61 % were receiving a single blockade, and 19 % a dual blockade. These proportions were 17, 72 and 11 % respectively for patients with a Pu < 1 g/day or an UPCR < 1 g/g at year two.

Controlled BP at year two, lowest baseline Pu values, antialdosterone treatment at year two and the absence of centrally acting antihypertensive drugs at baseline were significant factors associated with reaching the Pu target in the multivariate analysis (Fig. 4b). We didn’t observe any correlation with the use of single or dual RAS blockade.

### Metabolic parameters

HbA1C values were available for 504 patients at the end of the study. Glycemic control was overall stable after a 2-year follow-up (mean HbA1C 7.3 ± 1.2 %) with 37.7 % of the patients with HbA1C < 7 %, 38.1 % between 7 and 8 % and 24.2 % of patients above 8 %. At year two, 11 % of the patients were no longer receiving any antidiabetic treatment (vs 2 % at baseline).

LDL-cholesterol (LDL-c) values were reported for 326 patients after the 2-year follow-up and 64 % of them had a LDL-c value < 1 g/L.

### Renal events and eGFR decrease

**Fig. 3:**
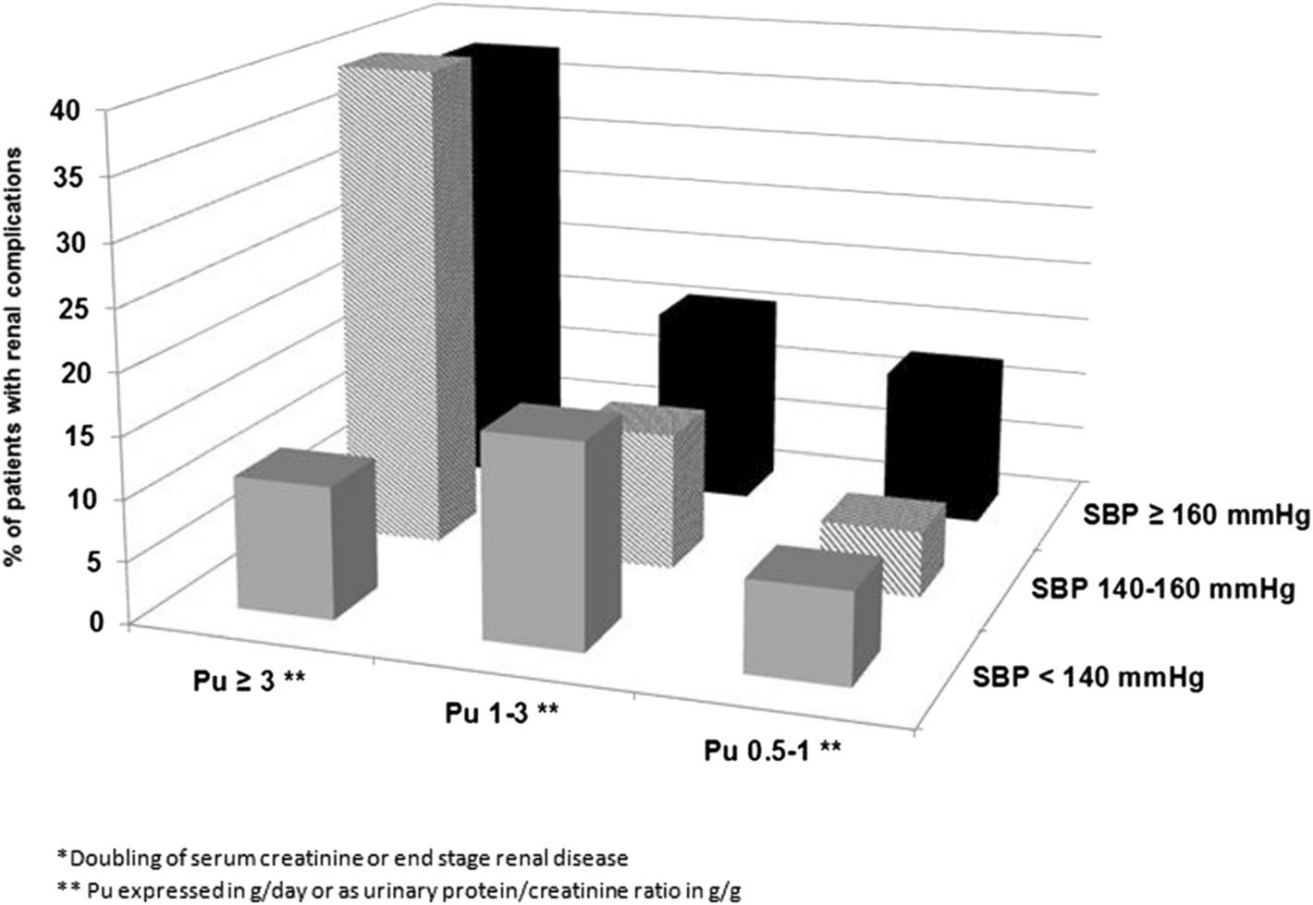
Renal complications * after a two year follow-up according to Pu and SBP levels at baseline

**Fig. 4:**
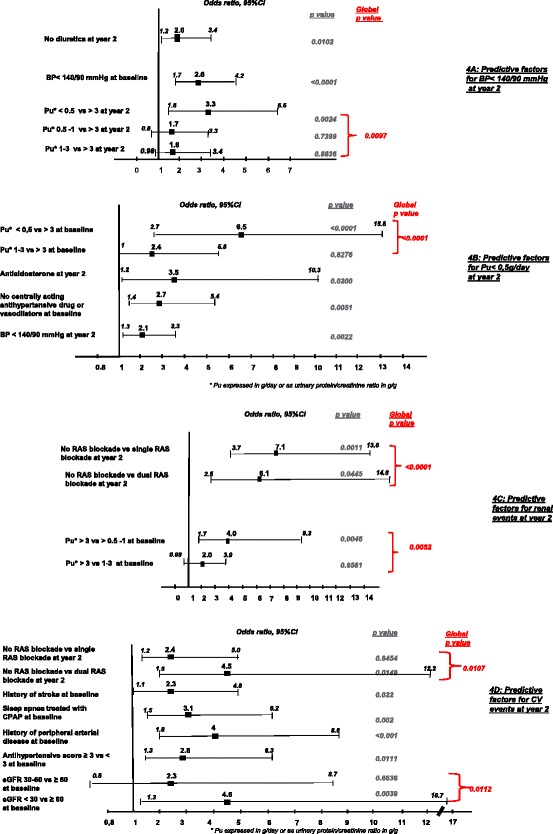
Associated factors with BP and Pu control and cardiovascular and renal events occurrence. **a** Associated factors BP <140/90 mmHg at year 2. The following parameters (significant in univariate analysis) were included in the model : smoking status, BP control at baseline, antihypertensive score at baseline and at year 2, prescription of CCB, diuretics, antialdosterone or centrally acting drugs at baseline and at year 2, Pu level and severity of renal failure at year 2, diabetes control and history of retinopathy at baseline. **b** Associated factors with Pu < 0,5 g/day at year 2. The following parameters (significant in univariate analysis) were included in the model : age, BP control at year 2, Pu level and severity of renal failure at baseline, prescription of antialdosterone or centrally acting drugs treatment at baseline and year two. **c** Associated factors with renal events at year 2. The following parameters (significant in univariate analysis) were included in the model : BP control at baseline, Pu level and severity of renal failure at baseline and at year 2, the antihypertensive treatment score at year two, RAS blockade at baseline and year 2, prescription of CCB, diuretics, centrally acting drugs or statins at baseline and at year 2. **d** Associated factors with cardiovascular events at year 2. The following parameters (significant in univariate analysis) were included in the model : age, duration of hypertension and diabetes, CPAP-treated sleep apnoea, severity of renal failure at baseline and year 2, LDL cholesterol control at baseline, antihypertensive treatment score at baseline, RAS blockade at year 2, prescription of antiplatelet drugs, CCB, diuretics, betablockers or centrally acting drugs at baseline and at year 2, history of CV disease at baseline (coronary heart disease, history of stroke, peripheral arterial disease of the lower extremities, or hospitalisation for heart failure)

During follow-up, 118 patients suffered from deterioration in renal function (16.2 %): doubling of serum creatinine for 86 patients (11.8 %) and/or start of dialysis treatment for 72 (9.9 %). None of the patients was transplanted. The incidence of these renal complications was higher in patients who had a medical history of CV disease at baseline (18.6 % of patients compared to 14.3 % with no history of CV disease at baseline). We also observed a correlation between deterioration of renal function, Pu rate and SBP levels at baseline (Fig. 3). In multivariate analysis the only predictors for the occurrence of renal complications were a higher Pu at baseline and the absence of treatment by one or two RAS blockers at year two, with no correlation observed with HbA1c level (Fig. 4c).

The mean eGFR of 40 ± 20.3 ml/min/1.73 m^2^ at baseline dropped to 36.5 ± 20.9 by year one and to 33.9 ± 22.6 ml/min/1.73 m^2^ by year two (*p* <0.001). This corresponds to a mean annual eGFR reduction of 3.2 ml/min/1.73 m^2^. The relative annual loss of renal function was 4.3 % for patients in stage 1 CKD, 8 % in stage 2, 7.2 % in stage 3a, 8.5 % in stage 3b and reached 11.3 % in stage 4. The GFR slope differed according to both the Pu and the BP level at baseline. The higher the Pu at baseline, the more marked was the decline in renal function: 5.3 % when Pu was between 0.5 and 1 g/day, 7.9 % when Pu was between 1 and 3 g/d and 13 % for Pu higher than 3 g/d. Similarly, the decline in renal function was more marked when BP was uncontrolled (> 140/90 mmHg) at baseline, 9.5 % versus 5.5 % when BP was controlled. This was despite the fact that the mean eGFR at baseline was similar in all these groups.

### Cardiovascular events and death

Out of the 729 patients for whom we had follow-up data at year two, 60 had died, i.e., 8.2 %: 23 during year one and 37 during year two. Twenty-six patients died from CV causes, 16 from another cause of death (no details available), and 18 from unknown causes. The baseline demographic and clinical data for these 60 patients did not differ greatly from the total population analysed at baseline (*n* = 986): they had a mean age of 74 years, an average eGFR of 37 ml/min/1.73 m^2^, 35 % of them suffered from severe renal failure and 53 % had a history of macrovascular complications.

One hundred seventy-six patients (24.1 %) developed at least one CV complication during the follow-up period, and among these, 50 also developed renal complications (doubling of serum creatinine and/or ESRD). Overall, 61 patients (8.4 %) had a coronary event (acute coronary syndrome and/or coronary revascularisation), 25 patients (3.4 %) had a stroke, 32 patients (4.4 %) underwent a lower limb revascularisation procedure, 17 patients (2.3 %) required amputation and 80 patients (11 %) were hospitalised for acute heart failure. The risk of developing such complications was twice as high among patients who had a medical history of CV complications at baseline (data not shown).

Among the patients who were free from CV complications at baseline (*n* = 412), the incidence of CV complications during the follow-up period increased in line with the severity of the CKD. For example, coronary heart disease was observed in 2.9 % of patients with an initial eGFR ≥ 60 ml/min/1.73 m^2^, 5.6 % in those with an eGFR between 30 and 60 ml/min/1.73 m^2^, and 7.5 % in those with an eGFR < 30 ml/min/1.73 m^2^. The incidence of heart failure requiring hospitalisation was 2.9, 6.2 and 10.2 % for these patient groups respectively and 5.7, 9.2 and 15 % for macrovascular complications (coronary heart disease, peripheral arterial disease, and stroke).

In multivariate analysis, the predictors of CV complications occurrence were a medical history of stroke, history of peripheral arterial disease in the lower extremities, sleep apnoea treated with continuous positive airway pressure (CPAP) at baseline, a lack of treatment with RAS blockers at year two, a higher antihypertensive treatment score and severe CKD at year two (Fig. 4d).

## Discussion

This large real-life study of patients with T2DM and CKD confirmed that BP and Pu targets remain a major challenge with only 11 % of the patients with BP < 140/90 mmHg and UPCR < 0.5 g/g after a 2-year follow-up. Furthermore the renal events rate was higher than the death rate.

### BP control

Less than 40 % of our patients had BP < 140/90 mmHg at the end of the2-year follow-up despite the use of 3 or more antihypertensive agents for 75 % of the patients at baseline, in line with what is usually reported in patients with impaired renal function [25]. Overall this lack of BP control could be explained by patient’s poor compliance, insufficient doses of diuretics, use of concomitant medications increasing BP, excessive salt intake, etc… [25] It is also well-known that BP control is more difficult as renal function worsens. Therapeutic inertia has also been found to play a role as described by Desai and al [26]. This retrospective study conducted in hypertensive patients with CKD showed that true therapeutic inertia accounted for 44 % of the patients who remained hypertensive while in the other 56 %, physicians either changed the medication regimen or documented clear reasons not to change the treatment or tried to change it. In our study, therapeutic inertia could be one of the explanations for the relatively high proportion of patients who remained hypertensive: over one third of the patients had a reduced treatment score irrespective of BP control at year 2.

However, these results must be interpreted with caution as BP was mainly monitored by office- based measurement, which has been demonstrated to be insufficient to classify CKD patients with hypertension [27]. In our study, HBPM or ABPM values were reported for less than 10 % of the patients despite relatively strong data demonstrating that in patients with CKD, BP measured at home is a better predictor of ESRD and CV events than BP measured in the office [28, 29].

### Pu control

In the same way, only 20 % of the patients followed in our study reached the Pu target after a 2-year follow-up. It has been largely demonstrated that on top of BP control, Pu control is also key to slow renal disease progression [30, 31]. This was also observed in our study, as we saw a correlation between deterioration in renal function and persistently high Pu rates and uncontrolled SBP. Unfortunately, we cannot compare our result with other large-scale “renal morbidity and mortality” interventional studies of diabetic patients with CKD and proteinuria as Pu targets are not reported as such [15, 32–34]. Considering the importance of Pu control, it is disappointing to note that changes over time in Pu were evaluable in only 72 % of the followed patients, either because the nephrologists did not report any measurement at the follow-up visit or because the Pu was only reported in concentration unrelated to 24-h urines or urinary creatinine.

### Use of RAS blockers

RAS blockers have proven to be effective in reducing Pu and slowing CKD progression beyond BP decrease in T2DM patients [15, 33] and are therefore recommended for all patients with T2DM and CKD [35].

However, the residual renal risk remains high and specific studies have been conducted to test the hypothesis of a better protection with a dual RAS blockade. In relatively short-term initial studies Pu control has been shown to be better with a dual RAS blockade than with a single agent [17, 36]. Unfortunately, more recently two long-term controlled studies (ALTITUDE & VA-NEPHRON) involving patients with T2DM and CKD reported that even if dual RAS blockade decreased Pu, not only did this not translate into any advantage in terms of CV and/or renal morbidity but it also led to an increase in renal complications (acute renal failure and hyperkaliemia) [32, 34]. The findings of ALTITUDE (conducted with the DRI aliskiren in dual RAS blockade) led the European Medicines Agency (EMA) to change aliskiren’ label introducing a contraindication for its use in patients with diabetes or renal failure in combination with ACEi or ARBs at the beginning of 2012. Given that the risk of adverse events from a dual RAS blockade exists independently of aliskiren use [32, 37, 38], the recent Pharmacovigilance Risk Assessment Committee recommendations on the EMA website have also drawn prescribers’ attention to the risk of adverse events in patients receiving a dual RAS blockade. Thus the labelling for all RAS blockers is progressively changing. At baseline, 23 % of our patients were on a dual RAS blockade, and in spite of changes in recommendations following ALTITUDE publication [34], a full 20 % were taking two RAS blockers after the 2-year follow-up, although aliskiren-based dual blockades had considerably decreased.

The proven efficacy of RAS blockade in T2DM and CKD patients explains why over 90 % of our patients were receiving at least one RAS blocker at baseline. What is more surprising is that about 20 % of patients were no longer receiving any RAS blockers after the 2-year follow-up, including those who still had high Pu values, and 20 % received a dual blockade. Similarly, a study conducted in US veterans with CKD found that the rate of ARBs/ACEi discontinuation was also high: less than 10 % of the patients remained on treatment throughout the whole all follow-up period [39]. The design of our study does not allow us to speculate about the reasons for discontinuing RAS blockers; for e.g., we do not have any information about the occurrence of severe hyperkaliemia or acute renal failure during the follow-up.

### Renal events and death

In our study CV events were frequent (12.1 % per year) and renal disease progression (8.5 % per year) was twice as high as the risk of death (4.3 % per year). Conversely, in several studies conducted in patients with CKD from all causes [40] as well as in diabetic patients with CKD [12], death was more than twice as likely as renal disease progression. This difference could be explained by improvements in the management and treatment of patients at high CV risk over the last 20 years.

As expected, the mortality rate in our study is higher than that reported in the Framingham study for diabetic patients during the period 1976–2001 (1.58 % per year), because of concomitant CKD [41]. However, it is lower than the mortality rate observed in the historical interventional studies IDNT and RENAAL (respectively 6 and 6.8 % per year). One might have thought that mortality would have been higher in a real life study, but once again this finding could be due to changes in management of these patients in the last 15 years [12, 15, 33]. The rate of renal events in our study exceeded those noted in the placebo arm of ALTITUDE [34] (2.1 % per year), probably because the mean eGFR at baseline was lower (40 vs 57 mL/min/1.73 m^2^) but also perhaps because involvement of patients in clinical trials modify disease progression maybe related to changes in physicians’ practice and/or patient compliance [42].

### Strengths and limitations

This analysis reports real life data and follow-up on a relatively large sample of a population with T2DM and advanced CKD for whom we have little information. The inclusion criteria in our study did not allow us to study the natural history of CKD from microalbuminuria to ESRD. One of the main limitations of the study is that 26 % of the originally enrolled and analysed patients were lost to follow-up at the end of year two but this is one of the known drawbacks of a real life study. Secondly, the data collected are purely observational and subject to declaration bias. Furthermore, as some results are lacking, we were not able to explore all parameters for all followed patients. However data in real-life setting may have broader applicability versus that from clinical trials with restrictive inclusion/exclusion criteria and strict monitoring of the practice.

## Conclusions

Our study highlights that achieving BP and Pu targets remains a major challenge in patients with T2DM and nephropathy in clinical practice, even when they are managed by nephrologists. Furthermore, our study shows that cardiovascular events and renal disease progression are more frequent than death unlike what has been observed in previous cohorts.

Our data also suggest that there is room for improvement especially in terms of standardization of BP monitoring by using ambulatory methods, treatment regimen and of Pu measurement.

## Abbreviations

ABPM: Ambulatory blood pressure monitoring
ACEi: Angiotensin converting enzyme inhibitors
ARB: Angiotensin 2 receptor blockers
BMI: Body mass index
BP: Blood pressure
CI: Confidence interval
CKD: Chronic kidney disease
CV: Cardiovascular
DBP: Diastolic blood pressure
DRI: Direct renin inhibitors
eGFR: estimated glomerular filtration rate
EMA: European medicines agency
ESRD: End stage renal disease
HBPM: Home blood pressure monitoring
MI: Myocardial infarction
OR: Odds ratio
Pu: Proteinuria
RAS: Renin angiotensin system
SBP: Systolic blood pressure
T2DM: Type 2 diabetes mellitus
UPCR: Urinary protein/creatinine ratio
